# Extinctions in Heterogeneous Environments and the Evolution of Modularity

**DOI:** 10.1111/j.1558-5646.2009.00684.x

**Published:** 2009-08

**Authors:** Nadav Kashtan, Merav Parter, Erez Dekel, Avi E Mayo, Uri Alon

**Affiliations:** 1 Department of Molecular Cell Biology, Weizmann Institute of ScienceRehovot, 76100 Israel; 2 E-mail: urialon@weizmann.ac.il

**Keywords:** Biological networks, extinction-recolonization, genetic recombinations, metapopulation, modularity

## Abstract

Extinctions of local subpopulations are common events in nature. Here, we ask whether such extinctions can affect the design of biological networks within organisms over evolutionary timescales. We study the impact of extinction events on modularity of biological systems, a common architectural principle found on multiple scales in biology. As a model system, we use networks that evolve toward goals specified as desired input–output relationships. We use an extinction–recolonization model, in which metapopulations occupy and migrate between different localities. Each locality displays a different environmental condition (goal), but shares the same set of subgoals with other localities. We find that in the absence of extinction events, the evolved computational networks are typically highly optimal for their localities with a nonmodular structure. In contrast, when local populations go extinct from time to time, we find that the evolved networks are modular in structure. Modular circuitry is selected because of its ability to adapt rapidly to the conditions of the free niche following an extinction event. This rapid adaptation is mainly achieved through genetic recombination of modules between immigrants from neighboring local populations. This study suggests, therefore, that extinctions in heterogeneous environments promote the evolution of modular biological network structure, allowing local populations to effectively recombine their modules to recolonize niches.

Extinctions occur on many scales. Mass extinctions that eliminate a large fraction of all existing life forms are fortunately rare (Raup 1986; Knoll 1989; Jablonski 1991; Raup 1993). More common are extinctions of individual species (Smith 1989; Raup 1994; Newman 1997). Reasons for the observed high frequency of species extinction are thought to include niche disappearance, and lack of ability to evolve rapidly enough to meet changing environments (Smith 1989; Bak and Sneppen 1993; Newman 1997).

The most common form of extinctions is probably that of localized subpopulations within a given species. Examples include extinctions of local populations of parasites when their host dies, a forest region when fire occurs, or a population of amphibians when a pool goes dry. Such an extinction of local populations is a recurrent rather than a unique event in the history of a species.

Local extinctions and their effect on population genetics have been extensively studied theoretically and experimentally. A useful conceptual framework for these studies is the metapopulations dynamics model, in which local populations evolve in separate localities (habitat patches) with heterogeneous local conditions (Hanski 1999). Individuals can migrate between neighboring localities. Local populations can go extinct, and then the free localities can be recolonized by immigrants from neighboring localities. Most studies of metapopulations focused on questions of genetic variability, biodiversity, and dynamics of populations (Maruyama and Kimura 1980; Ehrlich and Ehrlich 1981; Wright 1986; Slatkin 1987; Wade and McCauley 1988; McCauley 1991; Levin 1992; Tilman and Kareiva 1997; Hanski 1998, 1999; Pannell and Charlesworth 1999; Carlson and Edenhamn 2000; Kassen 2002; Tilman et al. 2002; Rousset 2004; Dey and Joshi 2006; Keymer et al. 2006). Here, we extend this to study the effect of local extinctions on internal organization—namely the structure of biological networks within organisms. This topic was rarely addressed (Wright 1986; Levin 1992; Raup 1994; Hanski and Heino 2003). We focus on modularity—a general structural property of biological systems.

Modularity is defined as the separability of the design into units that perform independently, at least to a first approximation (Wagner and Altenberg 1996; Hartwell et al. 1999; Lipson et al. 2002). Examples of modularity occur on all scales in biology. The body plan of organisms includes limbs and organs as modules, each with defined functions. Modularity also appears in the structure of biochemical networks within the cell: Signaling pathways, metabolic pathways, and coregulated gene groups are all modules of interacting molecules with a shared function and defined input and output ports. Modularity is even observed in the structure of many biomolecules (e.g., protein domains).

Although biological systems are commonly modular, a given system across different organisms may vary in its degree of modularity. An example that shows a range of modularity is metabolic networks. In previous work (Parter et al. 2007), we studied the degree of modularity of metabolic networks across over 100 bacterial species using modularity-assessing algorithms (Newman and Girvan 2004). We found that some bacterial species have metabolic networks that are highly modular, whereas others have nonmodular networks that cannot be separated into distinct metabolic modules (see Parter et al. [2007] for more information). Protein structure is another example for varying degrees of modularity. Many proteins are composed of defined structural domains such as regulatory sites, interaction domains, and catalytic sites, each with a specific function. Other proteins such as ribosomal proteins are less modular and cannot be readily decomposed into separated functional domains. Thus, modularity is not an inevitable feature but may be selected by evolution under certain conditions.

The evolutionary origin of modularity is particularly puzzling, because evolution in simulations almost always converges toward a nonmodular design (Kashtan and Alon 2005). The nonmodular designs are commonly selected in simulations due to the fact that modular designs are very rare and often less optimal than the nonmodular designs. Interestingly, even when the initial condition of the simulation is a modular design, evolution typically moves toward a nonmodular design that satisfies the same goal (Kashtan and Alon 2005).

Explanations for the emergence of modularity can be divided into two classes [reviewed in Callebaut and Rasskin-Gutman (2005) and Wagner et al. (2007)]. The first class suggests that modularity emerges as a result of a direct selective advantage such as selection for stability (Ancel and Fontana 2000; Variano et al. 2004), robustness (Thompson and Layzell 2000), or evolvability (Wagner and Altenberg 1996; Kirschner and Gerhart 1998; Gardner and Zuidema 2003; Hansen 2003; Sun and Deem 2007). In the second class of explanations, no direct selective advantage is associated with modularity, and instead modularity arises as a dynamical side effect of evolution. It has been suggested that modularity can emerge from duplications of subsystems or genes (Calabretta et al. 1998; Ward and Thornton 2007; Sole and Valverde 2008), subfunction fission (Force et al. 2005; Soyer 2007; Sole and Valverde 2008), or as a consequence of fluctuations (Guimera et al. 2004).

Here we address the origin of modularity by studying how extinctions in heterogeneous environments affect the modular structure of networks. We use computer simulations to evolve a well-studied computational model, networks composed of Boolean logic gates that evolve to compute Boolean functions. These model networks can be thought of a simple representation of several classes of biological networks such as cell signaling and gene regulation networks (Kashtan and Alon 2005). Such biological networks, similar to our model networks, have a set of input ports and a set of output ports and evolve to compute output values based on their inputs. For example, gene regulation networks compute responses (e.g., gene expression) to different signals (e.g., chemicals in the environment). Previous work on modularity showed that such logic circuits models can serve as representatives of a large class of simple evolutionary models including RNA structure and neural networks (Kashtan and Alon 2005; Kashtan et al. 2007).

To evolve model networks under spatially heterogeneous environments, we use the extinction–recolonization model. We thus evolve a metapopulation that occupies and migrates between multiple localities. The evolutionary goal is different in each locality. We build here upon the observation that environments in nature do not vary randomly from place to place, but rather seem to have common rules or regularities (Levins 1968; Kashtan and Alon 2005; Tagkopoulos et al. 2008). In this view, the goal of surviving in natural environment can be decomposed into a set of basic subgoals. Environmental conditions in a given locality pose a certain combination of subgoals. Consequently, organisms of different localities face challenges that share the same set of subgoals but in a different combination. We model this scenario by defining a distinct modular Boolean goal for each locality. Each such goal is a different combination of a given set of subgoals shared by all localities.

In previous studies (Kashtan and Alon 2005; Kashtan et al. 2007) we used a similar approach but without the spatial component—varying goals over time instead of space. This temporal variation denoted “modularly varying goals” (MVG) was found to promote the emergence of modular structure and to speed evolutionary rates. In contrast, the present study uses spatial variation in goals, rather than temporal variation, and is termed “spatial MVG” (SMVG) here. This leads to a new phenomena based on genetic recombinations that do not occur without the spatial component.

We find that in the absence of extinction events the evolved networks are highly optimal for their specific locality with a nonmodular structure. In contrast, under conditions in which extinction events occur, the evolved networks show a highly modular structure. The modular structure is selected because it allows organisms to adapt rapidly to the conditions of the free niche, and thus to colonize it following an extinction event. The rapid adaptation is mainly achieved by means of recombinations of modules between immigrants from neighboring localities.

## Results

We used a well-studied computational network model system, Boolean logic circuits. The circuits are composed of NAND (NOT-AND function) gates. Each circuit has several inputs (*x*, *y*, *z*, *w*) and a single output. The wiring of the gates was encoded in a genome made of a string of bits. The evolutionary goals were defined by Boolean functions. The goals were composed of XOR (exclusive-or), EQ (equal), and AND functions. The fitness of a circuit was defined as benefit minus cost. The benefit was given for the correctness of the desired computation and defined as the fraction of times the circuit gives the correct output, *G*, when evaluated over all possible Boolean values of the inputs. The cost was an increasing function of the number of logic gates in the network (see Methods).

**Figure 1:**
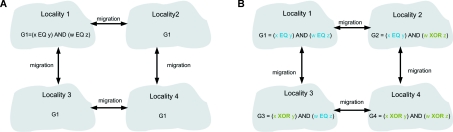
Extinction–migration model with local populations occupying four localities. Arrows represent migration routes between localities. The evolutionary goal in each locality is represented as a Boolean function G. (A) Homogeneous case (HG), where localities have identical goals (e.g., goal G1) (B) Heterogeneous case, where localities each have a different goal. Each goal was a different combination of the same set of subgoals (spatial modularly varying goals [SMVG]). In this example, the goals are an AND function on different combinations of XOR and EQ functions.

We applied standard genetic algorithms (Holland 1975; Goldberg 1989; Mitchell 1996) combined with a simple metapopulation model (Levins 1969; Hanski 1998) with four local populations occupying four localities. Each locality was associated with a specific goal (a Boolean function). Members of each local population migrated to neighboring localities with a given migration probability (see Methods) following a stepping stone model (Kimura and Weiss 1964) (Fig. 1).

In our simulations, we start with initial populations with random genomes. In every generation the fitness of each circuit is computed according to the goal in its current locality. Circuits with higher fitness have a higher probability to proliferate to the next generation. Recombinations (crossovers) and mutations were applied as genetic operators. The present results are insensitive to the number of localities, and to the migration and mutation rates (details of this insensitivity are given in a later section).

### CIRCUITS WITH A NONMODULAR STRUCTURE EVOLVE IN HOMOGENEOUS ENVIRONMENT WITH NO EXTINCTIONS

We begin with a scenario in which all localities have an identical goal (homogenous goal, abbreviated “HG”). For clarity, we will present results for one goal in detail, goal G1 = (*x* EQ *y*) AND (*w* EQ *z*) as described in Figure 1A, but the same conclusions are found for a wide range of goals as described below. The simulation started with random local populations of *N_pop_*= 1000 individuals occupying each of the four localities. Every generation a small fraction (*M_F_*= 0.1) of each local population migrated to neighboring localities. Through generations, fitness increased and after a few thousand generations the population in each of the localities reached maximal fitness.

**Figure 2:**
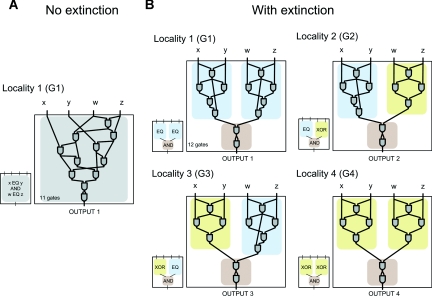
Structures of evolved circuits are nonmodular in HG and modular in SMVG with extinctions. (A) A typical circuit evolved without extinction in a homogeneous environment. Localities all had the same goal G1 = (*x* EQ *y*) AND (*w* EQ *z*). The circuit has a nonmodular structure. The gray gates represent NAND gates. Similar nonmodular circuits evolve also in the case in which localities had spatial modularly varying goals (SMVG) with no extinctions. (B) An example of circuits evolved when localities displayed spatial modularly varying goals (SMVG) with extinction events. The circuits are modular, with distinct structural modules for the EQ, XOR, and AND computations (shown in cyan, yellow, and brown respectively). The circuits evolved in each locality were composed of the combinations of modules that satisfied the local goal.

We analyzed the structure of the evolved circuits. We find that the evolved circuits had a nonmodular structure with a small number of gates (11 gates in the example, Fig. 2A). The circuits were highly optimal for the goal: they achieved the maximal benefit (solved the computation without errors) with a minimal cost (there exists no smaller circuit that solves G1).

**Figure 3:**
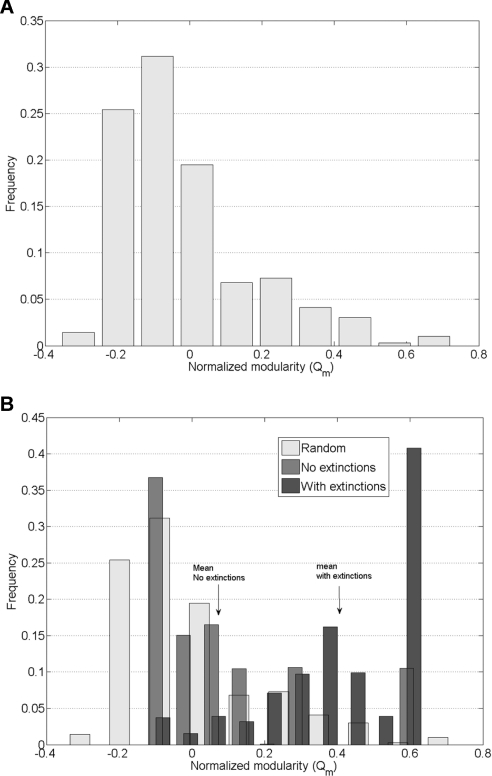
Circuits evolved under SMVG are more modular than most circuits that solve the same goals. (A) Normalized modularity measure (*Q_m_*) distribution of randomly sampled (nonevolved) circuits that perfectly solve the goal G4 = (*x* XOR *y*) AND (*w* XOR *z*). The distribution is for ∼300 circuits that solve the goal but were generated by an optimization algorithm rather than by an evolutionary process. (B) *Q_m_* distribution of evolved circuits that solve the goal G4. The circuits evolved under spatial modularly varying goals (SMVG), with and without extinctions (*E_x_*= 100).

**Figure 4:**
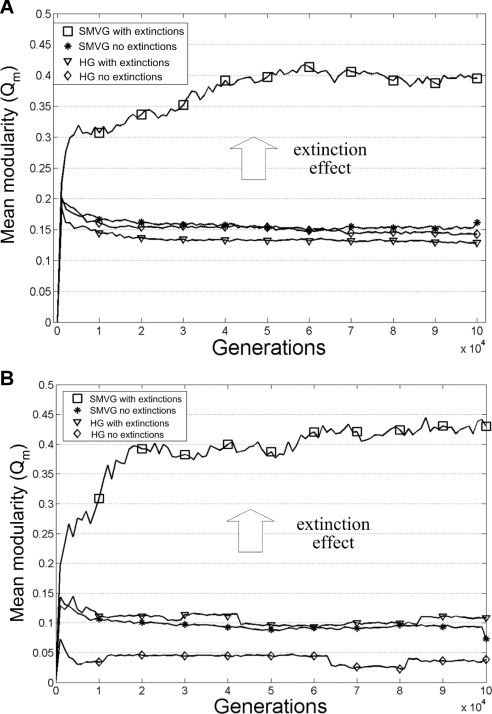
Modularity of evolved circuits as a function of generations. (A) Modularity through generations under four different scenarios. The graphs describe mean modularity measure, *Q_m_*, of all four local populations. (1) homogeneous localities (HG) with identical goals for all localities (G1–G4), no extinctions. (2) homogeneous localities (HG) with identical goals for all localities (G1–G4), with extinctions (*E_x_*= 100 generations). (3) heterogeneous localities with spatial modularly varying goals (SMVG composed of G1–G4), no extinctions. (4) heterogeneous localities with spatial modularly varying goals (SMVG composed of G1–G4), with extinctions (*E_x_*= 100 generations). *N_pop_*= 1000. Simulations included mutations and recombinations as genetic operators. Data are from 30 simulations for each of the scenarios. Error bars are smaller than the symbols on the lines. (B) Same as in (A) but for simulations without recombinations (asexual populations, with mutations only). *N_pop_*= 5000, *E_x_*= 20.

We repeated the simulations with three other HGs held same in all localities, G2–G4 defined in Figure 1B, that were composed of various combinations of XOR, EQ, and AND operations. We quantified the modularity of the evolved circuits using a network modularity measure, *Q_m_* (Newman and Girvan 2004; Kashtan and Alon 2005), (See Methods for details). Under this normalized measure, nonmodular networks are characterized by *Q_m_* close to zero, whereas modular networks typically show *Q_m_* values above 0.3. Note that for each of the goals G1–G4 there exist many different circuits that achieve the goal (solve it perfectly) with a wide range of *Q_m_* values (Fig. 3A). The circuits evolved toward any of the different spatially homogeneous goals G1–G4, had low values of this measure *Q_m_*= 0.15 ± 0.02 [mean ± SE] (Fig. 4A), indicating nonmodular solutions.

### EXTINCTIONS IN HOMOGENEOUS ENVIRONMENTS DO NOT INCREASE MODULARITY

Next, we evolved metapopulations under the same spatially homogeneous goals, but now with extinction events. An extinction event occurred every *E_x_*= 100 generations in a randomly chosen locality. When an extinction event occurred, the population of that locality was eliminated and immigrants from neighboring localities established the new population, competing for the empty locality. We find that the evolved circuit populations showed similar low modularity with *Q_m_*= 0.14 ± 0.02 (Fig. 4A). Thus, extinctions in a homogeneous environment did not have a significant impact on the modularity of the evolved circuits. This conclusion was found for a range of extinction rates *E_x_* spanning at least three decades.

### EXTINCTIONS IN A HETEROGENEOUS ENVIRONMENTS INCREASE MODULARITY

We next considered a situation in which localities had different goals as opposed to identical goals (see Fig. 1B), with SMVG. Each of the goals was composed of a different combination of the same set of subgoals: “EQ” (equal) and “XOR” (exclusive-or) functions (Fig. 1B). This set of MVG was identical to the set of goals discussed above in the homogeneous HG simulations, except that in the present case each locality had a different goal whereas in the HG case all localities had the same goal.

We repeated the simulations, now with SMVG localities, with and without extinction events. In the absence of extinction each local population evolved a different highly optimal circuit species that perfectly solved the goal of that locality with a minimal cost. The evolved populations showed low modularity *Q_m_*= 0.16 ± 0.02 (Figs. 2A, 3B, and 4A).

A striking difference was found when we considered extinction events in heterogeneous SMVG localities. We find that the circuit populations evolved in the presence of extinctions (*E_x_*= 100 generations) showed high modularity in all localities with *Q_m_*= 0.40 ± 0.03 (Figs. 3B and 4A). Examples of evolved circuits are shown in Figure 2B. The circuits evolved modules that correspond to the subgoals shared by the different varying goals: modules that compute XOR and EQ, combined with a module that computes AND.

The modular circuits were found to be suboptimal in terms of number of components. The circuits solve the goal perfectly, but on average they are larger by one gate, which is reflected in a mean reduction of 0.05 in their fitness (the fitness cost of an additional gate, see Methods). For example the modular circuits for G1 are now composed of 12 gates as opposed to 11 gates without extinctions, see Figure 2. In other words, modularity evolves despite its cost.

### MECHANISM FOR THE EMERGENCE OF CIRCUITS WITH A MODULAR STRUCTURE

Why do modular circuits evolve when we introduce extinctions in a heterogeneous environment? Whenever an extinction event occurs, a free niche is created (a free locality). Immigrants from neighboring localities then compete to colonize the locality. Because the goal of the free niche is a modular variation on the goals of the neighboring localities, circuits with a modular design can adapt rapidly to achieve the new goal. Thus, over many generations, nonmodular, highly adapted circuits will be selected against, or simply be eliminated by an extinction event. The modular circuits, in contrast, will be selected for their ability to rapidly fit the freed niches.

### ADAPTATION TO FREE NICHES IS ACHIEVED MAINLY BY GENETIC RECOMBINATION

**Figure 5:**
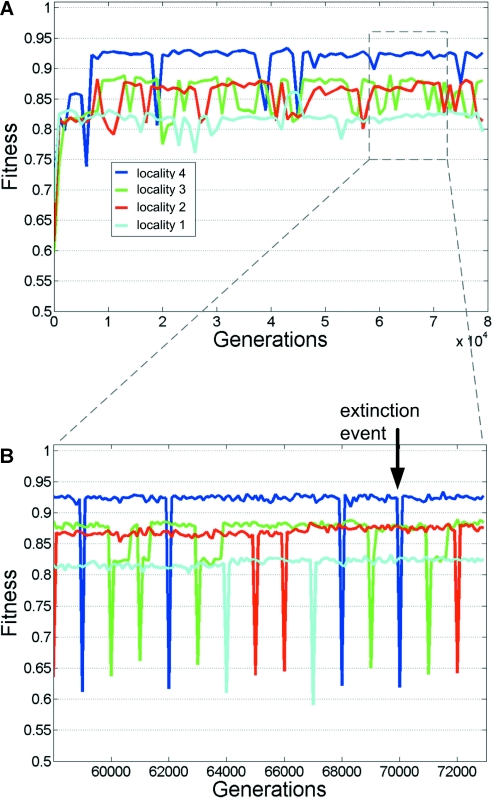
The fitness of the metapopulation along SMVG evolution with extinctions. (A) Mean fitness of each local population (shown in different colors) versus generations. (B) Zoom into fitness around several extinction events. Following an extinction event (arrow) the mean fitness in that locality initially drops, and then recovers rapidly. *E_x_*= 1000 generations, *N_pop_*= 1000.

In the present simulations, rapid adaptations occur mainly due to recombinations (crossovers) of two immigrants coming from two different neighboring localities. For example, following an extinction event in locality 1 (with goal G1), a recombination between a modular immigrant from locality 2 (that has the EQ module on the inputs *x* and *y*) and a modular immigrant from locality 3 (that has the EQ module on the inputs *w* and *z*) is likely to yield a high fitness circuit for G1 (Fig. 2B). In this way, the fitness of the immigrant population in a locality following an extinction event recovers rapidly. Typically, in only a few generations the new immigrants achieve mean fitness that is comparable to the fitness of the extinct previous population (Fig. 5).

### ANCESTRAL HISTORY IN SMVG TRANSITS BETWEEN FREED LOCALITIES

**Figure 6:**
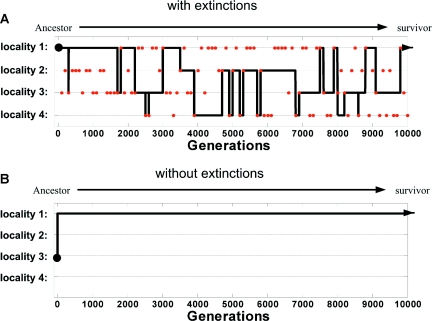
Ancestral history in SMVG evolution transits between freed niches. Shown is a typical trajectory of the localities where the ancestors of a survival circuit evolved through generations. Data are shown for the best circuit from locality 1 at the end of 10,000 generations simulation. Vertical lines represent migration events. Red points represent extinction events. (A) With extinctions. (B) Without extinctions. Simulation was without recombinations, to allow following a pure nondivergent evolutionary path. *E_x_*= 100 and *N_pop_*= 2500. Similar results were found with recombinations.

To better characterize the evolutionary process, we examined the ancestral history of individuals that survived evolution with SMVG and extinctions. We tracked the localities in which the ancestral line lived over the generations (Fig. 6). We find that the ancestral line repeatedly moved to new localities that had just undergone extinction (Fig. 6A). The probability of a successful ancestral migration to move to a just-extinct locality was 0.87 ± 0.03, which is far more often than expected at random (0.22 ± 0.02, *P* < 0.001).

A similar analysis in a simulation without extinctions showed that ancestors of survivors had a very low probability to have undergone migrations (Fig. 6B). On average, a successful migration in the ancestral line happened every more than 10,000 generations as opposed to every 260 ± 20 generations in a scenario with extinctions (*E_x_*= 100). Thus, survivors in a scenario with extinctions effectively undergo temporally varying goals, a scenario known to promote modularity (Kashtan and Alon 2005; Kashtan et al. 2007). In contrast, survivors in a scenario without extinctions effectively evolve under a constant, temporally unchanging goal (Fig. 6B).

### MODULARITY IS INCREASED UNDER A WIDE RANGE OF EXTINCTION AND MIGRATION RATES

**Figure 7:**
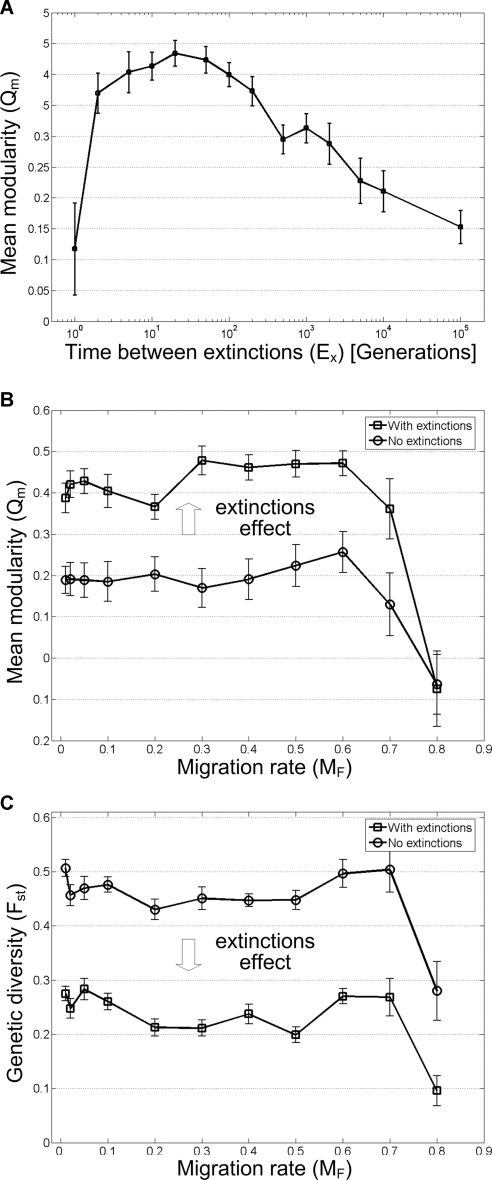
Effects of simulation parameters on modularity and genetic diversity. (A) The effect of extinction rate (*E_x_*) on modularity measure (*Q_m_*) in SMVG evolution. Each point represents the mean *Q_m_* (±SE) of the four local populations, *M_F_*= 0.1. (B) The effect of migration rate (*M_F_*) on modularity measure in the presence of extinctions (*E_x_*= 100) and without extinctions. For both (A) and (B) the mean is for 20 simulations of length 10^5^ generations, and *Q_m_* values were averaged over the last 3 × 10^4^ generations of each simulation. (C) The effect of migration rate (*M_F_*) on genetic diversity measure (*F*_ST_) of the metapopulation in the presence of extinctions (*E_x_*= 100) and without extinctions. *N_pop_*= 1000 for (A),(B) and (C). Data are of 20 simulations.

We also studied the effect of varying the model parameters. We evolved metapopulations with SMVG localities under various extinction rates from an extinction event every single generation, *E_x_*= 1, to an event every *E_x_*= 10^5^ generations (which is equivalent, in our simulations, to virtually no extinctions at all). We find that high modularity evolved over three orders of magnitude of extinction rates (Fig. 7A). After reaching a peak at around *E_x_*= 20 generations, the modularity measure *Q_m_* decreases with increasing times between extinctions in an approximately logarithmic manner.

What happens if extinction events are not periodic but rather a stochastic event? We simulated such a scenario with extinction events drawn randomly from a log-normal distribution with mean *E_x_*= 20 generations (SD =+180, −18). Circuits evolved under such a stochastic extinction scenario were found to be modular with *Q_m_*= 0.33 ± 0.03.

We find that modularity emerged, in the presence of extinctions, also under a wide range of migration rates (fraction of the population that migrates every generation). Modular circuits evolve from very low rates of *M_F_*= 0.01 to very high rates of *M_F_*= 0.6 (Fig. 7B). Under extremely high migration rates (*M_F_* > 0.7), the metapopulation typically does not achieve any of the goals, and very low modularity is found both with and without extinctions.

Finally, we asked what happens if migration rate is not constant but rather changes stochastically. Simulations of such a situation under SMVG with no extinctions with stochastic migration rates, drawn randomly from a log-normal distribution with mean *M_F_*= 0.1 (SD =+0.4, −0.08), yielded relatively low modularity *Q_m_*= 0.22 ± 0.03. Thus, even occasional extreme migration rates do not seem to be enough to generate modularity in the absence of extinctions.

### EXTINCTIONS IN HETEROGENEOUS ENVIRONMENTS REDUCE SPECIATION AMONG LOCALITIES

We further analyzed the impact of extinction events on the genetic diversity of the metapopulation evolved under SMVG. We first analyzed the genotypes of populations evolved without extinctions. We find that individuals from different localities had relatively distant genomes, differing in about 40% of their genomic positions (mean relative Hamming distance was HD = 0.4 ± 0.02). A standard measure for genetic diversity of metapopulations developed by Wright (1951) and later enhanced to fit genomic positions diversity by Nei (1973), shows a large diversity, *F*_ST_= 0.53 ± 0.01 (Table 1 and Methods). Interbreeding of individuals from two different localities (e.g., a recombination between a native individual and an immigrant) thus has a very low probability to survive. This is the reason that immigrants typically failed to compete with the native population of a given locality. Thus, each local population evolved into an independent species that was highly optimal to the locality goal.

**Table 1:** Modularity and genetic diversity are affected by extinctions in a heterogeneous environment. See Methods for definitions of HD_*s*_, HD_*tot*_ and *F*_ST_. Mean±SE is presented for all the measures for at least 30 independent simulations. *F*_ST_ had *P* <0.001 for all cases (*P*-value was computed using the permutation test [Raymond and Rousset 1995]).

Localities	Scenario	Modularity (*Q_m_*)	HD*_s_*	HD*_tot_*	*F*_ST_
With recombination					
Homogeneous (HG)	No extinctions	0.15±0.02	0.08±0.01	0.08±0.01	0.02±0.01
	Extinctions	0.14±0.02	0.12±0.02	0.12±0.02	0.00±0.01
Heterogeneous (SMVG)	No extinctions	**0.16±0.02**	0.16±0.01	0.33±0.01	**0.53±0.01**
	Extinctions	**0.40±0.03**	0.12±0.01	0.17±0.01	**0.28±0.01**
Without recombination					
Homogeneous (HG)	No extinctions	0.04±0.02	0.16±0.02	0.16±0.02	0.02±0.01
	Extinctions	0.11±0.02	0.12±0.02	0.12±0.02	0.02±0.01
Heterogeneous (SMVG)	No extinctions	**0.09±0.02**	0.16±0.01	0.36±0.01	**0.55±0.01**
	Extinctions	**0.42±0.03**	0.11±0.01	0.15±0.01	**0.25±0.02**

A different picture was observed in the presence of extinctions. We find that the genotypes from different SMVG localities were relatively similar, differing in only 20% of the genomic positions (mean Hamming distance [HD]= 0.2 ± 0.03). The positions that showed high variation between populations tended to encode for the specific gates that were rewired when a XOR module switched to an EQ module. The metapopulation showed much lower between-subpopulation variation (*F*_ST_= 0.28 ± 0.01) than in the lack of extinctions case (*F*_ST_= 0.53 ± 0.01). Indeed, interbreeding of individuals from different local populations was the main mode of adaptation to free niches following extinction. Thus, extinctions seem to preserve the metapopulation as a single highly adaptive species, with spatial allelic variations.

We also explored the impact of migration rates on genetic diversity. We find that in the presence of extinctions the impact of migration rate on *F*_ST_ is minor compared to Brown and Pavlovic (1992). This holds for a wide range of migration rates tested from *M_F_*= 0.01 to *M_F_*= 0.7 (Fig. 7C).

### CIRCUITS WITH A MODULAR STRUCTURE CAN EVOLVE ALSO IN THE ABSENCE OF RECOMBINATIONS, BUT ADAPTATION IS SLOWER

Finally, we asked whether the same results are observed in the absence of genetic recombinations, a case that corresponds to species with asexual reproduction (no recombinations between different members of the population). It also applies to the case in which each locality hosts a different species, which cannot interbreed. We find that the results hold also in this case of no recombinations: modularity emerges only if extinction events are introduced in heterogeneous localities (Fig. 4B).

Note that in this case, the mode of adaptation for free niches was mutations rather than recombinations. Adaptation was somewhat slower than in the case of recombinations (two to three generations were typically required for a first fully adapted organism to appear, as opposed to a single generation in the case with recombinations). Modular circuits evolved with the ability to rapidly adapt to the goals of their neighboring localities within one or two mutations that rewired a XOR module to an EQ module or vice versa (a similar mechanism to that described in Parter et al. [2008]). In summary, extinction events in asexual evolution is found to preserve the metapopulation as a single species with a modular structure with mutations as the key mode of adaptation.

## Discussion

The present study suggests that extinctions in heterogeneous environments can affect the design of networks within simple evolved computational “organisms”. Extinction events substantially increase modularity in heterogeneous environments that display spatial MVG. Through generations, the organisms sample recently extinct localities by migration and “learn” the set of subgoals shared by all localities. They are thus able to evolve a module for each of the subgoals. The modularity allowed a rapid adaptation to the niches freed by extinction events, by rewiring, or recombining modules to fit the goal in the free niche. Rapid adaptation to free niches is obtained predominantly by genetic recombinations (crossover, sex) between immigrants from two distinct neighboring localities. In addition, we find that in the absence of extinctions local populations eventually diverged into different species, each highly optimized for the conditions in its own locality, with a nonmodular structure. Extinctions reduce this tendency to speciation and lead to the emergence of a single species with a modular structure and spatial allelic variations.

How relevant are these results to natural evolution? Examples of metapopulations that spread over localities with heterogeneous conditions are very common in nature. A large fraction of species on earth are highly specific in their habitat requirements and live in rather separated local populations with extinctions–recolonizations dynamics (Wright 1986; Levin 1992; Tilman and Kareiva 1997; Hanski 1999). A well-studied example is of the Glanville fritillary butterfly (*Melitaea cinxia*) in the southwest of Finland (Hanski 1998, 1999). The butterfly persists in numerous, small, more-or-less isolated populations breeding on dry meadows. Extinction of local populations is a common event, typically followed by recolonization by new immigrants (Hanski 1998, 1999). There are two factors that have a major effect on the survival probability of the butterfly (1) the availability of a larval host plant and (2) the existence of parasitoids. There are two primary species of host plants and two primary types of parasitoids that attack the butterfly larva, and both factors vary widely between localities (Kankare et al. 2005). If adaptation to host plants and parasitoids can be considered as separate subgoals, the butterflies are faced with SMVG. The SMVG are composed of two subgoals, each of which can be one of two possibilities (analogous to the subgoals in our simulations). More generally, assuming that the niches in nature display variations on a set of biological subgoals, the present study suggests that organisms with modular networks will have an advantage in adapting to free niches more rapidly.

Several currently studied ecosystems may be used to empirically test the effects of local extinctions. These include forests with frequent fires (York 1999; Crawford et al. 2001) or sea-floor regions that are recurrently damaged by trawl-fishing (Engel and Kvitek 1998; Thrush and Dayton 2002). Trawling is a relevant example as it effectively causes local extinctions of sea-floor benthic communities (Engel and Kvitek 1998). Comparisons of sea-floor benthic communities between regions that were not trawled to ones that were frequently trawled indicated a significance decrease in biodiversity, a change in the community composition, and the increased abundance of fast-growing opportunistic species such as oligochaetes and nematodes (Engel and Kvitek 1998). These data provide evidence for the selection due to recurrent local extinctions. The selection pressures are similar to the ones we describe in our model: Local extinction may promote the survival of highly adaptive species that can rapidly fit into free niches following extinction events.

This work relates to the large literature on evolution in changing environments, beginning with the seminal work of Levins (1968). These studies tried to explore the interplay between environmental variation and the mechanisms for adaptation (Levins 1962, 1968; Lachmann and Jablonka 1996; Rainey and Travisano 1998; Meyers and Bull 2002). A significant body of literature studied the association between environmental variability and polymorphism (Levene 1953; Haldane and Jayakar 1963; Gillespie 1972; Frank and Slatkin 1990; Turelli et al. 2001), physiological adaptations (Schmalhausen 1949; Schlichting and Pigliucci 1998), probabilistic strategies (Cohen 1966; Bull 1987), developmental plasticity, and alternative phenotypes (Waddington 1953; Moran 1992). The impact of heterogeneous environments on population dynamic (Levin 1976) and the evolution of generalists and specialists (Van Tienderen 1991) was also extensively studied. The present study aims at understanding additional mechanism for coping with varying environment, namely the modular architecture of biological networks.

The present study extends our previous studies of modularity. In our previous study (Kashtan and Alon 2005), we showed that environment that changes with time can promote the evolution of modularity. In this study, we extend this by demonstrating how spatial variation can also promote modularity. Modularity is enhanced when spatial variation is combined with local extinctions. As shown in Figure 6, organisms evolved in spatially varying environment with extinctions effectively face temporally varying environments.

However there is an important difference between this study and our previous studies on temporally varying goals (Kashtan and Alon 2005). We find that in a population evolving under temporally varying goals, the primary means of adaptation to new goals was mutations. Adding recombination did not affect evolution. Here we studied variations in space rather than in time, so that several local populations evolved concurrently in several localities, each under a different goal. In contrast to (Kashtan and Alon 2005), we find that recombinations of genomes from different local populations are the primary means of adaptation to empty niches. The genetic diversity between subpopulations makes recombination effective, a diversity that is lacking in our previous studies that employed a single population. This highlights the usefulness of recombinations in metapopulations that evolve in heterogeneous environments with occasional extinctions. This view may add to the theory that aims to explain the prevalence of sexual reproduction (Maynard-Smith 1978; Kondrashov 1998; Otto and Lenormand 2002; Cooper 2007).

The present results may also be relevant for extinctions that eliminate an entire species rather than local populations, and even for the rare mass-extinctions of a large number of species, as is seen from our results of simulations without recombinations. The theory further predicts that the more rare the extinctions, the less modular the networks and the higher is the tendency to form specialized species. This prediction can in principle be tested by comparing modularity levels of biological networks of closely related organisms from natural environments with different extinction rates, as demonstrated in Parter et al. (2007) and Kreimer et al. (2008).

In summary, the present study demonstrates that extinctions may provide a selective pressure on the internal network structure of evolved organisms. Extinctions create free niches, and thus pose a selective pressure for designs that can rapidly adapt to these niches. If the free niches and their neighboring populated niches share subgoals, networks with a modular architecture tend to be selected over networks with a nonmodular architecture. Genetic recombination is a major driving force in this adaptation, by its power to recombine modules from neighboring local populations. It will be interesting to further study the impact of extinction on the design principles (Alon 2007) of biological molecules, networks, and organisms.

## Methods

### EVOLUTIONARY SIMULATIONS WITH LOGIC CIRCUITS MODEL

We used standard genetic algorithms to evolve Boolean logic circuits composed of two-inputs NAND gates. Each circuit corresponded to a binary genome that specified the connections between the gates, as described in (Kashtan and Alon 2005). The settings of the simulations were as follows: A population of *N_pop_* individuals was initialized to random binary genomes, in each of the four localities. The genomes were composed of 13 genes: 12 genes that coded for gate wiring and a single gene that coded for the output wiring (Kashtan and Alon 2005). In each generation, all the individuals in each local population were evaluated to compute their fitness *F*, defined as the fraction of all values of the inputs that produce the goal output minus the cost associated with the number of gates used in the circuit (see below). In each local population, pairs of circuits were chosen in accordance with their fitness, with probability proportional to exp(*t*×*F*) (*t*= 30 in our simulations), recombined (applying crossover operator), and randomly mutated (mutation probability *P_m_*= 0.7 per genome). Simulations lasted 10^5^ generations. The local-population size for the presented results with recombinations was *N_pop_*= 1000, and without recombinations *N_pop_*= 5000. The results hold also for larger local populations (sizes up to *N_pop_*= 50,000 were tested); the presented sizes were empirically determined to be the smallest to consistently yield the present results. Qualitatively similar results were observed also with different mutation rates, selection strategies (e.g., different *t* in the selection probability, or using an elite selection strategy (Vasconcelos et al. 2001)) and different modular goals (replacing AND with OR functions, goals with six inputs as in (Kashtan et al. 2007)).

### FITNESS CALCULATION

Fitness of a circuit is defined as *F*=δ−η, where δ (the benefit) is equal to the fraction of all possible input values for which the circuit gives the desired output. The cost η is defined as the number of effective gates in the circuit (gates with a path to the output) above a predefined number of gates (for the presented results 10 gates, penalty was 0.05 for each additional gate).

### MIGRATION

Migrations were performed as described by the arrows in Figure 1. Every generation, emigrants were chosen randomly from each local population. The number of emigrants was *M_F_*= 0.1 of the population size. Different values of *M_F_* ranging from *M_F_*= 0.01 to *M_F_*= 0.7 were tested and yielded qualitatively similar results. Migration destination was randomly chosen according to the migration graph (Fig. 1). After each migration event, to keep the population size fixed, population was selected proportionally to fitness. Qualitatively similar results were obtained using two different migration rules: (1) emigrants were selected randomly with dependency on fitness—individuals with median fitness had higher probability to emigrate. (2) Emigrants were selected randomly from the population without dependency on fitness.

### EXTINCTIONS

Extinction events eliminated a local population chosen at random and occurred every *E_x_* generations. Each extinction event was followed by a recolonization by migrations from neighboring localities. For simplicity, we considered a fixed local-population size. In “just” freed niches, immigrants were expanded to the full local-population size by proportional selection with replacements as described above.

### POPULATION GENETIC SUBDIVISION MEASURE

*F*_ST_ measure (Wright 1951; Nei 1973; Raymond and Rousset 1995) was computed by: *F*_ST_= (HD*_tot_*− HD*_s_*)/(HD*_tot_*), where HD*_tot_* is the relative number of positions by which two genomes from the metapopulation are different (mean relative HD). HD_s_ is the same measure for a single subpopulation. Low *F*_ST_ values (close to zero) indicate that the genetic diversity within and between subpopulations is similar, suggesting that all localities represent a single species. High *F*_ST_ values (0.3–1) indicate that genetic diversity between subpopulations is much higher than within the subpopulations, suggesting different species.
